# Finite-Time Dynamic Tracking Control of Parallel Robots with Uncertainties and Input Saturation

**DOI:** 10.3390/s21092996

**Published:** 2021-04-24

**Authors:** Mengyang Ye, Guoqin Gao, Junwen Zhong, Qiuyue Qin

**Affiliations:** School of Electrical and Information Engineering, Jiangsu University, Zhenjiang 212013, China; 2111707008@stmail.ujs.edu.cn (M.Y.); 2211807055@stmail.ujs.edu.cn (J.Z.); 2111807010@stmail.ujs.edu.cn (Q.Q.)

**Keywords:** parallel robot, finite-time control, input saturation, auxiliary system, nonsingular terminal sliding mode, disturbance observer

## Abstract

This paper considers the finite-time dynamic tracking control for parallel robots with uncertainties and input saturation via a finite-time nonsingular terminal sliding mode control scheme. A disturbance observer is designed to estimate the lumped disturbance in the dynamic model of the parallel robot, including modeling errors, friction and external disturbance. By introducing the fractional exponential powers into the existing asymptotic convergent auxiliary system, a novel finite-time convergent auxiliary system is constructed to compensate for input saturation. The finite-time nonsingular terminal sliding mode control is proposed based on the disturbance estimation and the state of the novel auxiliary system, so that the convergence performance, control accuracy and robustness are improved. Due to the estimation and compensation for the lumped disturbance, the inherent chattering characteristic of sliding mode control can be alleviated by reducing the control gain. The finite-time stability of the closed-loop system is proved with Lyapunov theory. Finally, simulation and experimental research on the dynamic control of a conveying parallel robot are carried out to verify the effectiveness of the proposed method.

## 1. Introduction

Due to the mechanical properties of strong carrying capacity, high precision and fast response speed, parallel robots exhibit outstanding performance in various applications, e.g., industry, agriculture, medical equipment [1,2,3]. However, the system complexity, purposeful simplification of the system dynamics and circumstance variations introduce uncertainties to the dynamic control of parallel robots, such as modeling errors, friction and external disturbance, all of which can be defined as the lumped disturbance. Moreover, the control input saturation caused by inherent physical constraints of the actuator also affects the dynamic control performance and the stability of the system. Therefore, the dynamic tracking control of parallel robots with uncertainties and input saturation is investigated in this paper.

Sliding mode control (SMC) [4] exhibits strong robustness against bounded uncertainties acting on the control channel. In recent years, an extended adaptive fuzzy SMC method for the position control of Stewart parallel manipulator can be found in [5]. A second-order SMC method for cable-driven parallel robots was presented in [6]. Since the linear sliding surface is applied in [5,6], only the asymptotic convergence of the system state can be ensured. In contrast, the terminal SMC (TSMC) based on the nonlinear sliding surface [7] is an effective finite-time control strategy. To overcome the singularity of the TSMC, the nonsingular TSMC (NTSMC) was proposed for the tracking control of rigid manipulators [8]. Thereafter, research about improved versions of NTSMC was further presented in [9,10]. Recent studies on the finite-time tracking control of uncertain robot systems via NTSMC can be seen in [11,12,13] and the references therein. However, the impact of input saturation on the control performance is not considered in [11,12,13].

In addition to uncertainties, input saturation also occurs in practical control systems when the control input command exceeds the maximum output of the actuator. The resulting difference between the actual saturation input and the input command is known as the input saturation error, which should not be ignored. Otherwise, the control performance will be degraded and, even worse, the stability of the control system will be destroyed [14,15]. This issue was considered in [16,17], where the input saturation error was regarded as a part of the composite disturbance, which was estimated by a disturbance observer. Nevertheless, the calculation burden of the designed observer in [16,17] would be increased and the system dynamic characteristics can also be affected by the extra disturbance derived from the input saturation. The smooth hyperbolic tangent function was adopted in [18,19,20] to approximate the saturation function in the actual saturation control input, thus eliminating the sharp corner in the saturation function. In [21,22,23], SMC was used as the master controller with other assistive technology to deal with the uncertainties and input saturation in system. Recently, a strategy to cope with the input saturation problem by constructing an auxiliary system can be found in [24,25,26,27,28,29,30,31,32,33,34,35,36,37,38,39,40]. The basic principle is to take the input saturation error as the input of the designed auxiliary system, and the control law is then designed based on the state variables of the auxiliary system. However, it should be noted that in the above-mentioned literature [16,17,18,19,20,21,22,23,24,25,26,27,28,29,30,31,32,33,34,35,36,37,38,39,40], the tracking errors can only be convergent to a neighborhood of the origin asymptotically, which is very different from finite-time convergence to the origin.

In terms of the dynamic control of parallel robots, finite-time control techniques can provide faster convergence speed, higher control precision and better anti-disturbance performance [41]. Therefore, the aim of this study is to design a finite-time dynamic tracking control strategy for parallel robots with uncertainties and input saturation. A disturbance observer is first designed to estimate the lumped disturbance composed of modeling errors, friction and external disturbance in system. By introducing the fractional exponential powers into the existing auxiliary system [24,25,26,27,28,29,30,31,32,33,34] with asymptotic convergence, a novel finite-time auxiliary system is then designed to compensate for the input saturation. Compared with the existing auxiliary system designed in [24,25,26,27,28,29,30,31,32,33,34], the novel designed auxiliary system including fractional exponential powers can be combined with the NTSMC algorithm to realize the finite-time control of parallel robots. Based on the disturbance estimation and the state of the novel auxiliary system, a finite-time nonsingular terminal sliding mode control (FT-NTSMC) for parallel robots is proposed. The contributions of this research are as follows:

1. In contrast to [16,17], the effect of input saturation is not mixed with the uncertainties, so that the additional disturbance caused by input saturation error is eliminated and the burden of disturbance observer is relieved;
2. In contrast to the existing control algorithm based on an asymptotically convergent auxiliary system [25,26,27,28,29,30,31,32,33], the proposed FT-NTSMC can realize the finite-time tracking control performance. Specifically, a novel finite-time convergent auxiliary system including fractional exponential powers is constructed to compensate for input saturation. The FT-NTSMC for parallel robots is attained by combining a disturbance observer-based NTSMC algorithm with the novel auxiliary system. Consequently, the finite-time convergence of both the sliding variable and the tracking error are ensured for parallel robots even in the presence of uncertainties and input saturation;
3. The sign function is not explicitly included in the proposed control law. Moreover, due to the estimation and compensation of the lumped disturbance, the control gain merely needs to be larger than the upper bound of the disturbance estimation error. Thus, the chattering of the proposed controller can be effectively attenuated.

The outline of this paper is organized as follows. Section 2 gives the preliminaries and the problem formulation. The controller design and the stability analysis are provided in Section 3. Simulation and experimental results based on the prototype system of a conveying parallel robot are respectively illustrated in Section 4 and Section 5. Section 6 is the discussion and Section 7 concludes the paper.

For convenience, the mathematical notations in the text are listed as follows:

Notation 1. For a *n*-dimensional vector $x={\left(x_{1},x_{2},\cdots ,x_{n}\right)}^{T}$, we have $x^{\alpha }=(x_{1}^{\alpha },x_{2}^{\alpha },\cdots ,$$x_{n}^{\alpha }{)}^{T}$, ${\left|x\right|}^{\alpha }={\left[{\left|x_{1}\right|}^{\alpha },\cdots ,{\left|x_{n}\right|}^{\alpha }\right]}^{T}$, $\mathrm{sign}\left(x\right)={\left[\mathrm{sign}\left(x_{1}\right),\cdots ,\mathrm{sign}\left(x_{n}\right)\right]}^{T}$, $\mathrm{sig}{\left(x\right)}^{\alpha }=$${\left[{\left|x_{1}\right|}^{\alpha }\mathrm{sign}\left(x_{1}\right),\cdots ,{\left|x_{n}\right|}^{\alpha }\mathrm{sign}\left(x_{n}\right)\right]}^{T}$.

Notation 2. ∥x∥ and ∥P∥ are the 2-norm of the vector x and the matrix P, respectively.

Notation 3. $\lambda_{\min}\left(P\right)$ and $\lambda_{\max}\left(P\right)$ denote the minimum and maximum eigenvalues of the matrix P, respectively.

## 2. Preliminaries and Problem Formulation

### 2.1. Dynamic Modeling

The dynamic model of n-DOF parallel robots can be formulated as

(1) $$\left[M(q)+\Delta M(q)\right]\ddot{q}+\left[C\left(q,\dot{q}\right)+\Delta C\left(q,\dot{q}\right)\right]\dot{q}+\left[G(q)+\Delta G(q)\right]+N\left(t\right)=\tau +\tau_{d},$$

where $q,\dot{q},\ddot{q}\in R^{n}$ denote the vectors of joint position, velocity and acceleration, respectively. $M\left(q\right)\in R^{n\times n}$, $C\left(q,\dot{q}\right)\in R^{n\times n}$, $G\left(q\right)\in R^{n}$ are the nominal parts of the positive definite inertial matrix, the centripetal Coriolis matrix and the gravitational vector, respectively. ΔM(q), $\Delta C(q,\dot{q})$, ΔG(q) stand for the unknown modeling errors, N(t) represents the friction, $\tau_{d}$ is the external disturbance and τ is the joint torque input vector.

Considering the multi-branch closed-chain characteristic of the parallel robot and the actual operating environment, the dynamic model of the parallel robot is complex and difficult to establish precisely. Specifically, the system complexity, purposeful simplification of the system dynamics and circumstance variations introduce uncertainties to the dynamic control of parallel robots, such as modeling errors, friction and external disturbance, which can be defined as the lumped disturbance. Therefore, the dynamic model (1) can be rewritten as

(2) $$\ddot{q}\left(t\right)=M^{-1}\left[\tau -C\left(q,\dot{q}\right)\dot{q}-G\left(q\right)\right]+D\left(t\right),$$

where $D\left(t\right)=M^{-1}[\tau_{d}-\Delta M\left(q\right)\ddot{q}-\Delta C\left(q,\dot{q}\right)\dot{q}-\Delta G\left(q\right)$−N(t)] represents the lumped disturbance term for convenience, including modeling errors, friction and external disturbance. The following assumption is imposed on the lumped disturbance D(t).

**Assumption** **1.** *There exist two positive constants d and $\bar{d}$, such that the lumped disturbance and its derivative satisfy the inequalities ∥D(t)∥≤d and $∥\dot{D}\left(t\right)∥\leq \bar{d}$, respectively.*

### 2.2. Problem Formulation and Control Objective

Besides the effect of lumped disturbance, input saturation is also ineluctable in practical control systems due to the physical limitations of actuators or the security constraints. When the input command exceeds the upper bound of the actuator output, the actual input signal is not enough to overcome the lumped disturbance in system, so that the control performance and system stability are damaged.

In this paper, the actual control input is denoted as $\tau ={\left[\tau_{1},\tau_{2},\cdots ,\tau_{n}\right]}^{T}$, which is a saturation function of the control input command $\tau_{c}={\left[\tau_{c1},\tau_{c2},\cdots ,\tau_{cn}\right]}^{T}$, i.e.,

(3) $$\tau_{i}=\mathrm{sat}\left(\tau_{ci}\right)=\left\{\begin{array}{cc} \tau_{i\max}, & \tau_{ci}>\tau_{i\max}\\ \tau_{ci}, & \tau_{i\min}\leq \tau_{ci}\leq \tau_{i\max},\;i=1,2,\cdots ,n,\\ \tau_{i\min}, & \tau_{ci}<\tau_{i\min}, \end{array}\right.$$

where $\tau_{i\max}>0$ and $\tau_{i\min}<0$ are the known upper and lower bound of the control input, respectively. There is a difference between the actual input signal τ and the input command $\tau_{c}$, which is defined as the input saturation error $\tilde{\tau }=\tau -\tau_{c}$.

To compensate for the input saturation, an existing auxiliary system with asymptotic convergence was proposed in [25,26,27,28,29,30,31,32,33]

(4) $$\dot{\bar{\zeta }}=\left\{\begin{array}{c} -B\bar{\zeta }-\frac{\left|S^{T}M^{-1}\tilde{\tau }\right|+0.5{\tilde{\tau }}^{T}\tilde{\tau }}{∥\bar{\zeta }{∥}^{2}}\bar{\zeta }+\tilde{\tau },\;∥\bar{\zeta }∥\geq \zeta_{0},\\ 0,∥\bar{\zeta }∥<\zeta_{0}, \end{array}\right.$$

where $\bar{\zeta }$ is the auxiliary variable, B is a positive definite matrix, S denotes the linear sliding variable in terms of SMC, and $\zeta_{0}$ is a positive constant. However, the system tracking errors in [25,26,27,28,29,30,31,32,33] only asymptotically converge to a neighborhood of the origin.

**Remark** **1.** *To achieve finite-time control, the NTSMC with a nonlinear sliding surface is an effective scheme. However, when dealing with the input saturation problem, the existing auxiliary system (4) is difficult to combine with the NTSMC. Hence, the finite-time control of parallel robots with uncertainties and input saturation is still a challenging problem.*

The aim of this study is to design a FT-NTSMC algorithm, based on the disturbance estimation technique and a novel finite-time auxiliary system, for parallel robots with uncertainties and input saturation, so as to enable the actual trajectory q to track the desired trajectory $q_{d}$ in a finite-time, i.e., the tracking error $e=q-q_{d}$ will converge to the origin in a finite-time.

It should be noted that the inherent discontinuous switching characteristics of SMC will cause the chattering. Specifically, the sign function usually exists in the SMC law. The imperfection in the sign-function implementation yields a finite amplitude and finite frequency “zigzag” motion in the sliding mode due to the discrete-time nature of the computer simulation [4]. This effect is called “chattering”, which may induce fatigue in mechanical parts and even instability of the system. In this paper, the inherent chattering characteristic of SMC can be reduced by the proposed FT-NTSMC method.

### 2.3. Useful Lemmas

Finally, we give some lemmas that will be used throughout the article.

**Lemma** **1 ([42]).** *For any $x_{i}\in R,i=1,2,\ldots ,n$, 0<a≤1, one has*

(5) $${\left(\left|x_{1}\right|+\cdots +\left|x_{n}\right|\right)}^{a}\leq {\left|x_{1}\right|}^{a}+\cdots +{\left|x_{n}\right|}^{a}.$$

**Lemma** **2 ([17]).** *(Rayleigh-Ritz theorem) The Hermite matrix P and the vector x satisfy the inequality*

(6) $$\lambda_{\min}{\left(P\right)∥x∥}^{2}\leq x^{T}Px\leq \lambda_{\max}\left(P\right){∥x∥}^{2}.$$

**Lemma** **3 ([26]).** *For bounded initial conditions, if there exists a $C^{1}$ continuous and positive definite Lyapunov function V(x) satisfying $\varphi_{1}(∥x∥)\leq V\left(x\right)\leq \varphi_{2}(∥x∥)$, such that $\dot{V}\left(x\right)\leq -\varpi V\left(x\right)+\varepsilon$, where $\varphi_{1}$ and $\varphi_{2}$ are class K functions, ϖ and ε are positive constants, then the solution x(t) is uniformly bounded.*

**Lemma** **4 ([43]).** *Consider a dynamical system*

(7) $$\left\{\begin{array}{c} \dot{x}=f\left(x\right),\\ x\left(0\right)=x_{0},f\left(0\right)=0, \end{array}\right.$$

*where $x\in R^{n}$ is the state vector and f:$R^{n}\to R^{n}$ is a continuous vector field. If there exist μ>0,α∈(0,1) and a $C^{k}$ positive definite function V(x) such that $\dot{V}\left(x\right)\leq -\mu V^{\alpha }\left(x\right)$, the equilibrium point x=0 of system (7) is finite-time stable and the settling time is given by $t\leq \frac{V^{1-\alpha }\left(x_{0}\right)}{\mu (1-\alpha )}$.*

## 3. Design of FT-NTSMC Strategy for Parallel Robots

**Figure 1:**
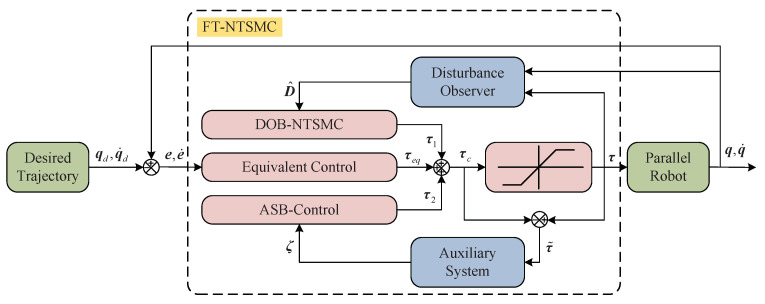
The schematic diagram of the FT-NTSMC for parallel robots.

In this section, based on a disturbance observer and a novel auxiliary system with finite-time convergence, the FT-NTSMC is proposed to deal with the issue discussed in Remark 1. The control schematic diagram is given in Figure 1.

As can be seen from Figure 1, the designed control input command $\tau_{c}$ consists of three parts. Specifically, $\tau_{1}$ is the disturbance observer-based NTSMC (DOB-NTSMC) term including the disturbance estimation $\hat{D}$, which is used to compensate for the lumped disturbance, so that the system states can reach the nonsingular terminal sliding surface in a finite-time; $\tau_{2}$ is the auxiliary system-based control (ASB-Control) term including the auxiliary variable ζ, which is used to compensate for the input saturation; $\tau_{eq}$ is the equivalent control to ensure that the system state can maintain the sliding mode motion and finally converge to the origin along the sliding surface in a finite-time.

### 3.1. Design of the Disturbance Observer

First, a disturbance observer [44] is designed to estimate the lumped disturbance D(t)

(8) $$\left\{\begin{array}{cc} \dot{Z} & =-LZ-L\left[M^{-1}\left(\tau -C\left(q,\dot{q}\right)\dot{q}-G\left(q\right)\right)+L\dot{q}\right],\\ \hat{D} & =Z+L\dot{q}. \end{array}\right.$$

where $\hat{D}$ is the disturbance estimation, Z is the state of the observer, $L=\mathrm{diag}(l_{1},l_{2},\cdots ,l_{n})$ is the observer gain and $L-\frac{I}{2}$ is a positive definite matrix. The result of disturbance observer (8) is given as follows.

**Lemma** **5.** *If the disturbance observer (8) is adopted to estimate the lumped disturbance in (2) and Assumption 1 is held, the disturbance estimation error $\tilde{D}\left(t\right)=D\left(t\right)-\hat{D}\left(t\right)$ will stable to the compact set $\Omega =\left\{\tilde{D}:∥\tilde{D}∥\leq \bar{d}/\lambda_{\min}\left(L\right)\right\}$ in a finite-time.*

**Proof** **of Lemma 5.** Considering (2) and (8), the derivative of $\hat{D}$ is formulated as

(9) $$\begin{array}{cc} \dot{\hat{D}} & =\dot{Z}+L\ddot{q}\\ \; & =-LZ-L\;\cdot \;L\dot{q}+LD\\ \; & =-L\hat{D}+LD\\ \; & =L\tilde{D}. \end{array}$$

Select the Lyapunov function as $V\left(\tilde{D}\right)=\frac{1}{2}{\tilde{D}}^{T}\tilde{D}$. It can be derived from (9), Lemma 2 and Assumption 1 that

(10) $$\begin{array}{cc} \dot{V}\left(\tilde{D}\right) & ={\tilde{D}}^{T}\dot{\tilde{D}}\\ \; & ={\tilde{D}}^{T}\left(\dot{D}-L\tilde{D}\right)\\ \; & =-{\tilde{D}}^{T}L\tilde{D}+{\tilde{D}}^{T}\dot{D}\\ \; & \leq -\lambda_{\min}\left(L\right){∥\tilde{D}∥}^{2}+\bar{d}∥\tilde{D}∥\\ \; & \leq -∥\tilde{D}∥\left(\lambda_{\min}\left(L\right)∥\tilde{D}∥-\bar{d}\right). \end{array}$$

It should be noted that if $\tilde{D}\in R^{n}\backslash \Omega$, i.e., $∥\tilde{D}∥>\bar{d}/\lambda_{\min}\left(L\right)$, we have $\dot{V}\left(\tilde{D}\right)<0$. Therefore, the disturbance estimation error will stabilize to the compact set Ω in a finite-time. □

**Remark** **2.** *Since $\lambda_{\min}\left(L\right)=\min\left\{l_{1},\ldots ,l_{n}\right\}$, $\bar{d}/\lambda_{\min}\left(L\right)$ can be selected as small as possible if the diagonal matrix L is appropriately chosen, so that $\tilde{D}\left(t\right)$ can converge to an any small compact set.*

### 3.2. Design of the Novel Auxiliary System

To deal with the issue discussed in Remark 1, a novel auxiliary system with finite-time convergence, which can be combined with the NTSMC, is constructed to compensate for input saturation. First, a nonsingular terminal sliding mode surface is designed as follows [9]

(11) $$s=e+\beta \mathrm{sig}{\left(\dot{e}\right)}^{\alpha }=0,$$

where $\beta =\mathrm{diag}(\beta_{1},\beta_{2},\cdots ,\beta_{n})$ is a positive definite diagonal matrix, and 1<α<2.

**Remark** **3.** *The time taken for the reaching phase of SMC is denoted as $t_{r}$, i.e., $s(t_{r})=0$. During the sliding mode phase, the time taken from $e_{i}\left(t_{r}\right)\neq 0$ to $e_{i}\left(t_{r}+t_{s}\right)=0$ is $t_{s}=\beta_{i}^{1/\alpha }\frac{\alpha }{\alpha -1}{\left|e_{i}\left(t_{r}\right)\right|}^{(\alpha -1)/\alpha }$, where $e_{i}$ represents the i-th component of the tracking error e.*

In contrast to the auxiliary system (4), a novel auxiliary dynamic system with finite-time convergence is constructed to compensate for the input saturation

(12) $$\dot{\zeta }=\left\{\begin{array}{cc} -\gamma_{1}\zeta -\gamma_{2}\zeta^{p_{1}/q_{1}}-\frac{\left|\eta M^{-1}\tilde{\tau }\right|+0.5\gamma_{3}{\tilde{\tau }}^{T}\tilde{\tau }}{{∥\zeta ∥}^{2}}\zeta +\gamma_{3}\tilde{\tau }, & ∥\zeta ∥>\zeta_{0},\\ 0, & ∥\zeta ∥\leq \zeta_{0}, \end{array}\right.$$

where ζ is the auxiliary variable, $\eta =s^{T}\beta \mathrm{diag}\left(|\dot{e}{|}^{\alpha -1}\right)$, $\gamma_{1},\gamma_{2},\gamma_{3},\zeta_{0}$ are positive constants, $p_{1}$ and $q_{1}$ are positive odd integers with $1/2<p_{1}/q_{1}<1$.

**Remark** **4.** *By incorporating the fractional exponential powers $\zeta^{p_{1}/q_{1}}$ and $\mathrm{diag}\left(|\dot{e}{|}^{\alpha -1}\right)$, the novel auxiliary system (12), which is finite-time convergent, is designed to compensate for the input saturation. Compared with the traditional auxiliary system (4), the novel auxiliary system (12) can be combined with the finite-time control approach.*

### 3.3. Design of the Proposed FT-NTSMC

For parallel robots with uncertainties and input saturation, based on the disturbance estimation $\hat{D}$ and the auxiliary variable ζ, the FT-NTSMC is designed in this section.

The dynamics of the sliding variable s is first given below

(13) $$\begin{array}{cc} \dot{s} & =\alpha \beta \mathrm{diag}\left(|\dot{e}{|}^{\alpha -1}\right)\ddot{e}+\dot{e}\\ \; & =\alpha \beta \mathrm{diag}\left(|\dot{e}{|}^{\alpha -1}\right)\left\{M^{-1}\left[\tau_{c}+\tilde{\tau }-C\left(q,\dot{q}\right)\dot{q}-G\left(q\right)\right]+D\left(t\right)-{\ddot{q}}_{d}\right\}+\dot{e}. \end{array}$$

Then, the FT-NTSMC law is designed as follows

(14) $$\left\{\begin{array}{c} \tau =\mathrm{sat}\left(\tau_{c}\right),\\ \tau_{c}=\tau_{1}+\tau_{2}+\tau_{eq}, \end{array}\right.$$

with

(15) $$\tau_{1}=-M\frac{\eta^{T}}{{∥\eta ∥}^{2}}∥s∥\rho k_{0}-M\frac{\eta^{T}}{{∥\eta ∥}^{2}}{∥s∥}^{2p_{2}/q_{2}}k_{1}-M\frac{\eta^{T}}{{∥\eta ∥}^{2}}{∥s∥}^{2}-M\hat{D},$$

(16) $$\tau_{2}=-MK_{2}\zeta -MK_{3}\eta^{T},$$

(17) $$\tau_{eq}=M{\ddot{q}}_{d}+C\left(q,\dot{q}\right)\dot{q}+G\left(q\right)-\alpha^{-1}M\beta^{-1}\mathrm{sig}{\left(\dot{e}\right)}^{2-\alpha },$$

where $\rho =∥\beta \mathrm{diag}\left(|\dot{e}{|}^{\alpha -1}\right)∥$, the positive integers $p_{2}$ and $q_{2}$ satisfy $p_{2}/q_{2}=\left(p_{1}+q_{1}\right)/2q_{1}$, $k_{0}>\bar{d}/\lambda_{\min}\left(L\right)$, $k_{1}>0$. The positive definite diagonal matrices $K_{2}$ and $K_{3}$ should be properly chosen such that $K_{3}-\frac{K_{2}}{2}$, $\gamma_{1}I-\frac{\gamma_{3}}{2}I-\frac{K_{2}}{2}$ are also positive definite matrices.

### 3.4. Stability Analysis

The main result of the proposed FT-NTSMC scheme is given in the following theorem.

**Theorem** **1.** *For the dynamic model of parallel robots (2), based on the disturbance observer (8) and the novel auxiliary dynamic system (12), if the FT-NTSMC law (14)–(17) is adopted, the nonsingular terminal sliding variable s and the tracking error e will converge to the origin in a finite-time.*

**Proof** **of Theorem 1.** *Step 1:* Select a Lyapunov function as

(18) $$V_{1}=\frac{1}{2}s^{T}s+\frac{\alpha }{2}\zeta^{T}\zeta +\frac{1}{2}{\tilde{D}}^{T}\tilde{D}.$$

Substituting (14)–(17) into (13), we have

(19) $$\begin{array}{cc} s^{T}\dot{s} & =\alpha s^{T}\beta \mathrm{diag}\left(|\dot{e}{|}^{\alpha -1}\right)\left\{M^{-1}\left[\tau_{c}+\tilde{\tau }-C\left(q,q\right)\dot{q}-G\left(q\right)\right]+D\left(t\right)-{\ddot{q}}_{d}\right\}+s^{T}\dot{e}\\ \; & =\alpha \eta \left[-\frac{\eta^{T}}{{∥\eta ∥}^{2}}{∥s∥}^{2p_{2}/q_{2}}k_{1}-\frac{\eta^{T}}{{∥\eta ∥}^{2}}∥s∥\rho k_{0}-\frac{\eta^{T}}{{∥\eta ∥}^{2}}{∥s∥}^{2}-K_{3}\eta^{T}-K_{2}\zeta +M^{-1}\tilde{\tau }+\tilde{D}\left(t\right)\right]\\ \; & =-\alpha k_{1}{∥s∥}^{2p_{2}/q_{2}}-\alpha {∥s∥}^{2}-\alpha ∥s∥\rho k_{0}+\alpha \eta \tilde{D}-\alpha \eta K_{3}\eta^{T}-\alpha \eta K_{2}\zeta +\alpha \eta M^{-1}\tilde{\tau }. \end{array}$$

Taking the derivative of $V_{1}$ and substituting (9), (12) and (19) produces

(20) $$\begin{array}{cc} {\dot{V}}_{1}= & s^{T}\dot{s}+\alpha \zeta^{T}\dot{\zeta }+{\tilde{D}}^{T}\dot{\tilde{D}}\\ = & -\alpha k_{1}{∥s∥}^{2p_{2}/q_{2}}-\alpha {∥s∥}^{2}-\alpha ∥s∥\rho k_{0}+\alpha \eta \tilde{D}-\alpha \eta K_{3}\eta^{T}-\alpha \eta K_{2}\zeta +\alpha \eta M^{-1}\tilde{\tau }\\ \; & +\alpha \left(-\gamma_{1}\zeta^{T}\zeta -\gamma_{2}\zeta^{T}\zeta^{p_{1}/q_{1}}-\left|\eta M^{-1}\tilde{\tau }\right|-0.5\gamma_{3}{\tilde{\tau }}^{T}\tilde{\tau }+\gamma_{3}\zeta^{T}\tilde{\tau }\right)+{\tilde{D}}^{T}\left(\dot{D}-L\tilde{D}\right). \end{array}$$

According to Lemma 5, one has $∥\tilde{D}∥\leq \bar{d}/\lambda_{\min}\left(L\right)$, so the following inequality holds

(21) $$\eta \tilde{D}\leq ∥\eta ∥∥\tilde{D}∥\leq \rho ∥s∥∥\tilde{D}∥\leq \rho ∥s∥\bar{d}/\lambda_{\min}\left(L\right).$$

The following inequalities can be directly deduced from Young’s inequality [27]

(22) $$-\eta K_{4}\zeta \leq \frac{1}{2}\eta K_{2}\eta^{T}+\frac{1}{2}\zeta^{T}K_{2}\zeta .$$

(23) $$\zeta^{T}\tilde{\tau }\leq \frac{1}{2}\zeta^{T}\zeta +\frac{1}{2}{\tilde{\tau }}^{T}\tilde{\tau }.$$

Substituting Equations (21)–(23) into Equation (20) yields

(24) $$\begin{array}{cc} {\dot{V}}_{1}\leq & -\alpha k_{1}{∥s∥}^{2p_{2}/q_{2}}-{\alpha ∥s∥}^{2}-\alpha ∥s∥\rho k_{0}+\alpha \rho ∥s∥\bar{d}/\lambda_{\min}\left(L\right)-\alpha \eta K_{3}\eta^{T}\\ \; & +\alpha \left(\frac{1}{2}\eta K_{2}\eta^{T}+\frac{1}{2}\zeta^{T}K_{2}\zeta \right)-\alpha \left(\gamma_{1}-\frac{\gamma_{3}}{2}\right)\zeta^{T}\zeta -\alpha \gamma_{2}\zeta^{T}\zeta^{p_{1}/q_{1}}+{\tilde{D}}^{T}\left(\dot{D}-L\tilde{D}\right)\\ \; & \leq -\alpha k_{1}{∥s∥}^{2p_{2}/q_{2}}-\alpha {∥s∥}^{2}-\alpha \rho ∥s∥\left(k_{0}-\bar{d}/\lambda_{\min}\left(L\right)\right)-\alpha \eta \left(K_{3}-\frac{K_{2}}{2}\right)\eta^{T}\\ \; & -\alpha \zeta^{T}\left(\gamma_{1}I-\frac{\gamma_{3}}{2}I-\frac{K_{2}}{2}\right)\zeta -\alpha \gamma_{2}\zeta^{T}\zeta^{p_{1}/q_{1}}+{\tilde{D}}^{T}\dot{D}-{\tilde{D}}^{T}L\tilde{D}. \end{array}$$

Please note that ${∥s∥}^{2p_{2}/q_{2}}⩾0$, $k_{0}>\bar{d}/\lambda_{\min}\left(L\right)$, $\zeta^{T}\zeta^{p_{1}/q_{1}}⩾0$; $K_{3}-\frac{K_{2}}{2}$ and $L-\frac{I}{2}$ are positive definite symmetric matrices, according to Lemma 2 and Assumption 1, we have

(25) $$\begin{array}{cc} {\dot{V}}_{1} & \leq -\alpha {∥s∥}^{2}-\alpha \zeta^{T}\left(\gamma_{1}I-\frac{\gamma_{3}}{2}I-\frac{K_{2}}{2}\right)\zeta -{\tilde{D}}^{T}L\tilde{D}+{\tilde{D}}^{T}\dot{D}\\ \; & \leq -\alpha s^{T}s-\alpha \zeta^{T}\left(\gamma_{1}I-\frac{\gamma_{3}}{2}I-\frac{K_{2}}{2}\right)\zeta -{\tilde{D}}^{T}L\tilde{D}+\frac{1}{2}{\tilde{D}}^{T}\tilde{D}+\frac{1}{2}{\dot{D}}^{T}\dot{D}\\ \; & \leq -\alpha s^{T}s-\alpha \lambda_{\min}\left(\gamma_{1}I-\frac{\gamma_{3}}{2}I-\frac{K_{2}}{2}\right)\zeta^{T}\zeta -\lambda_{\min}\left(L-\frac{I}{2}\right){\tilde{D}}^{T}\tilde{D}+\frac{1}{2}{\bar{d}}^{2}\\ \; & \leq -2\mu_{1}V_{1}+\frac{1}{2}{\bar{d}}^{2}, \end{array}$$

where $\mu_{1}=\min\left\{\alpha ,\lambda_{\min}\left(\gamma_{1}I-\frac{\gamma_{3}}{2}I-\frac{K_{2}}{2}\right),\lambda_{\min}\left(L-\frac{I}{2}\right)\right\}>0$.

It can be deduced from (25) that $0\leq V_{1}\left(t\right)\leq \frac{{\bar{d}}^{2}}{4\mu_{1}}+\left[V_{1}\left(0\right)-\frac{{\bar{d}}^{2}}{4\mu_{1}}\right]e^{-2\mu t}\leq V_{1}\left(0\right)+\frac{{\bar{d}}^{2}}{4\mu_{1}}$. According to Lemma 3, s, ζ, $\tilde{D}$ is globally uniformly ultimately bounded.

*Step 2:* Now we will prove the nonsingular terminal sliding variable s will converge to the origin in a finite-time. The Lyapunov function is selected as

(26) $$V_{2}=\frac{1}{2}s^{T}s+\frac{\alpha }{2}\zeta^{T}\zeta .$$

Taking the derivative of $V_{2}$ and substituting (21)–(23) yields

(27) $$\begin{array}{cc} {\dot{V}}_{2}= & s^{T}\dot{s}+\alpha \zeta^{T}\dot{\zeta }\\ = & -\alpha k_{1}{∥s∥}^{2p_{2}/q_{2}}-\alpha {∥s∥}^{2}-\alpha ∥s∥\rho k_{0}+\alpha \eta \tilde{D}-\alpha \eta K_{3}\eta^{T}-\alpha \eta K_{2}\zeta +\alpha \eta M^{-1}\tilde{\tau }\\ \; & +\alpha \left(-\gamma_{1}\zeta^{T}\zeta -\gamma_{2}\zeta^{T}\zeta^{p_{1}/q_{1}}-\left|\eta M^{-1}\tilde{\tau }\right|-0.5\gamma_{3}{\tilde{\tau }}^{T}\tilde{\tau }+\gamma_{3}\zeta^{T}\tilde{\tau }\right)\\ \leq & -\alpha k_{1}{∥s∥}^{2p_{2}/q_{2}}-\alpha {∥s∥}^{2}-\alpha ∥s∥\rho k_{0}+\alpha \rho ∥s∥\bar{d}/\lambda_{\min}\left(L\right)-\alpha \eta K_{3}\eta^{T}\\ \; & +\alpha \left(\frac{1}{2}\eta K_{2}\eta^{T}+\frac{1}{2}\zeta^{T}K_{2}\zeta \right)-\alpha \left(\gamma_{1}-\frac{\gamma_{3}}{2}\right)\zeta^{T}\zeta -\alpha \gamma_{2}\zeta^{T}\zeta^{p_{1}/q_{1}}\\ \; & \leq -\alpha k_{1}{∥s∥}^{2p_{2}/q_{2}}-\alpha {∥s∥}^{2}-\alpha \rho ∥s∥\left(k_{0}-\bar{d}/\lambda_{\min}\left(L\right)\right)-\alpha \eta \left(K_{3}-\frac{K_{2}}{2}\right)\eta^{T}\\ \; & -\alpha \zeta^{T}\left(\gamma_{1}I-\frac{\gamma_{3}}{2}I-\frac{K_{2}}{2}\right)\zeta -\alpha \gamma_{2}\zeta^{T}\zeta^{p_{1}/q_{1}}. \end{array}$$

Please note that ${∥s∥}^{2}⩾0$, $k_{0}>\bar{d}/\lambda_{\min}\left(L\right)$; $K_{3}-\frac{K_{2}}{2}$ and $\gamma_{1}I-\frac{\gamma_{3}}{2}I-\frac{K_{2}}{2}$ are positive definite symmetric matrices, thus we have

(28) $${\dot{V}}_{2}\leq -\alpha k_{1}{∥s∥}^{2p_{2}/q_{2}}-\alpha \gamma_{2}\zeta^{T}\zeta^{p_{1}/q_{1}}.$$

Reviewing Lemma 1, let $a=\left(p_{1}+q_{1}\right)/2q_{1}$, $\left|x_{i}\right|=\zeta_{i}^{2},i=1,\cdots ,n$, one has

(29) $${\left(\zeta_{1}^{2}+\cdots +\zeta_{n}^{2}\right)}^{\left(p_{1}+q_{1}\right)/2q_{1}}\leq \zeta_{1}^{\left(p_{1}+q_{1}\right)/q_{1}}+\cdots +\zeta_{n}^{\left(p_{1}+q_{1}\right)/q_{1}},$$

thus

(30) $${∥\zeta ∥}^{\left(p_{1}+q_{1}\right)/q_{1}}\leq \zeta^{T}\zeta^{p_{1}/q_{1}}.$$

Please note that

(31) $$-\alpha k_{1}{∥s∥}^{2p_{2}/q_{2}}=-2^{p_{2}/q_{2}}\alpha k_{1}{\left[{\left(\frac{∥s∥}{\sqrt{2}}\right)}^{2}\right]}^{p_{2}/q_{2}}=-2^{p_{2}/q_{2}}\alpha k_{1}{\left(\frac{1}{2}s^{T}s\right)}^{p_{2}/q_{2}}.$$

(32) $$-\alpha \gamma_{2}{∥\zeta ∥}^{\left(p_{1}+q_{1}\right)/q_{1}}=-2\gamma_{2}{\left(\frac{\alpha }{2}\right)}^{\left(q_{1}-p_{1}\right)/2q_{1}}{\left(\frac{\alpha }{2}\zeta^{T}\zeta \right)}^{\left(p_{1}+q_{1}\right)/2q_{1}}.$$

Combining (30)–(32), and noting that the parameters in the control law (14)–(17) satisfy $p_{2}/q_{2}=\left(p_{1}+q_{1}\right)/2q_{1}$, then inequality (28) is transformed to

(33) $$\begin{array}{cc} {\dot{V}}_{2} & \leq -\alpha k_{1}{∥s∥}^{2p_{2}/q_{2}}-\alpha \gamma_{2}{∥\zeta ∥}^{\left(p_{1}+q_{1}\right)/q_{1}}\\ \; & =-2^{p_{2}/q_{2}}\alpha k_{1}{\left(\frac{1}{2}s^{T}s\right)}^{p_{2}/q_{2}}-2\gamma_{2}{\left(\frac{\alpha }{2}\right)}^{\left(q_{1}-p_{1}\right)/2q_{1}}{\left(\frac{\alpha }{2}\zeta^{T}\zeta \right)}^{\left(p_{1}+q_{1}\right)/2q_{1}}\\ \; & \leq -\mu_{2}V_{2}^{p_{2}/q_{2}}, \end{array}$$

where $\mu_{2}=\min\left\{2^{p_{2}/q_{2}}\alpha k_{1},2\gamma_{2}{\left(\frac{\alpha }{2}\right)}^{\left(q_{1}-p_{1}\right)/2q_{1}}\right\}$.

According to Lemma 4, the nonsingular terminal sliding mode variable s will converge to the origin in a finite-time $t_{r}$ with $t_{r}\leq \frac{V_{2}^{1-p_{2}/q_{2}}\left(0\right)}{\mu_{2}\left(1-p_{2}/q_{2}\right)}$.

According to Remark 3, after reaching the nonsingular terminal sliding surface, the system states will continue to move along the sliding surface until they converge to the origin. Therefore, the convergence time of the tracking error $e_{i}$ from the initial state $e_{i}\left(0\right)$ to the origin is $T=t_{r}+t_{s}\leq \frac{V_{2}^{1-p_{2}/q_{2}}\left(0\right)}{\mu_{2}\left(1-p_{2}/q_{2}\right)}+\beta_{i}^{1/\alpha }\frac{\alpha }{\alpha -1}{\left|e_{i}\left(t_{r}\right)\right|}^{(\alpha -1)/\alpha }$. □

**Remark** **5.** *It should be noted that the sign function is not explicitly included in the control law (14)–(17). Moreover, the proposed control algorithm can estimate and compensate the lumped disturbance, the control gain $k_{0}$ merely needs to be larger than the upper bound of the disturbance estimation error $\bar{d}/\lambda_{\min}\left(L\right)$. According to Remark 2, if the matrix L is selected reasonably, $k_{0}$ can be chosen as small as possible, which is effective for chattering attenuation.*

**Remark** **6.** *To suppress the chattering of SMC, the boundary layer method that uses saturation function instead of sign function in the SMC law is a simple and common method. However, to reduce the chattering, the thickness of the boundary layer should be larger, resulting in poor control performance. Moreover, the boundary layer method can only ensure that the system states converge to a boundary layer centered on the sliding surface. Therefore, the sliding variable and the tracking error only converge to a neighborhood of the origin. In contrast, as proved in Theorem 1, the proposed FT-NTSMC can ensure the sliding variable and the tracking error converge to the origin in a finite-time. The chattering attenuation ability of FT-NTSMC has been explained in Remark 5. The following numerical simulation results and analysis can also verify the superiority of the proposed FT-NTSMC scheme.*

## 4. Numerical Simulation

To verify the proposed control method, numerical simulations are conducted on a 3-DOF conveying parallel robot (CPR) used for automobile electro-coating under three comparative controllers, i.e., the proposed FT-NTSMC, the disturbance observer-based NTSMC without auxiliary system (labeled as Controller-1) and the SMC with the traditional asymptotically convergent auxiliary system (4) (labeled as Controller-2).

Controller-1 based on the disturbance observer (8) is designed as

(34) $$\tau_{v1}=M{\ddot{q}}_{d}+C\left(q,\dot{q}\right)\dot{q}+G\left(q\right)-\alpha^{-1}M\beta^{-1}\mathrm{sig}{\left(\dot{e}\right)}^{2-\alpha }-M\frac{\eta^{T}}{{∥\eta ∥}^{2}}∥s∥\rho k_{0}-M\hat{D}.$$

Controller-2 based on the traditional asymptotically convergent auxiliary system (4) is designed as

(35) $$\left\{\begin{array}{c} \tau_{v2}=\mathrm{sat}\left(\tau_{c}\right),\\ \tau_{c}=-M\left(e-{\ddot{q}}_{d}+A\dot{e}+P\left(\bar{s}-\bar{\zeta }\right)+K\mathrm{sgn}\left(\bar{s}\right)\right)+C\left(q,\dot{q}\right)q+G\left(q\right), \end{array}\right.$$

where $\bar{s}=\dot{e}+Ae$ is a linear sliding variable, $\bar{\zeta }$ is the state variable of system (4); A, P and B−0.5P−0.5I are positive definite diagonal matrices, $K=\mathrm{diag}(k_{1},k_{2},\cdots ,k_{n})$ with $k_{i}>d\left(i=1,2,\cdots ,n\right)$.

**Figure 2:**
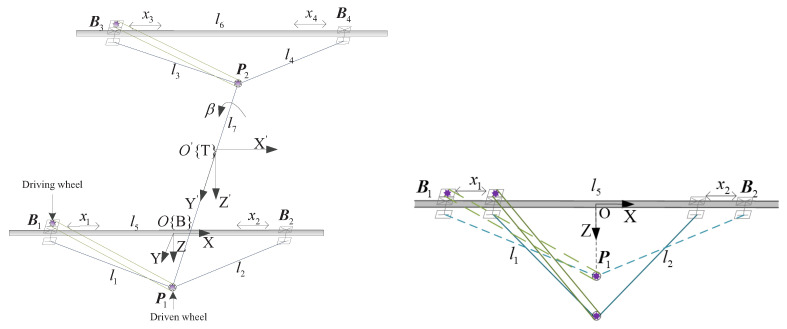
Structural sketch and side view of the CPR.

The structural sketch and side view of the CPR are illustrated in Figure 2. The physical parameters are listed in Table 1. As illustrated in Figure 2, the CPR exhibits a bilateral symmetrical structure with six active joints, i.e., four sliders (labeled as Joint 1∼Joint 4) responsible for translational motion and two driving wheels (labeled as Joint 5 and Joint 6) responsible for rotational motion.

The friction term [7] can be expressed as $N\left(t\right)=F_{c}\mathrm{sign}\left(\dot{q}\right)+B_{c}\dot{q}$ with $F_{c}=\mathrm{diag}$ (0.8, 0.8, 0.8, 0.8, 0.8, 0.8) being the Coulomb friction and $B_{c}=\mathrm{diag}\left(1,1,1,1,1,1\right)$ being the coefficient of the viscous friction. To verify the robustness of the proposed method, the modeling error is set to ΔM=diag(0.5,0.5,0.5,0.5,0.5,0.5) and the external disturbance is chosen as $\tau_{d}={\left[\tau_{d1},\tau_{d2},\cdots ,\tau_{d6}\right]}^{T}$ with $\tau_{d1}=\sin t+\cos2t$, $\tau_{d2}=\sin t-\cos2t$, $\tau_{d3}=-\sin t+\cos2t$, $\tau_{d4}=-\sin t-\cos2t$, $\tau_{d5}=\cos t+\sin2t$, $\tau_{d6}=\cos t-\sin2t$.

In all tests, the control aim is to regulate the active joints of the CPR to follow the desired trajectory. Controller-1 can be directly attained by removing the auxiliary system from the proposed FT-NTSMC law, and input saturation is not imposed on Controller-1. By the comparison of the proposed controller and Controller-1, the compensation ability to input saturation of the proposed controller can be verified. Controller-2 is chosen to demonstrate that the proposed FT-NTSMC can achieve better tracking performance and chattering attenuation ability. After simulation debugging, when the control performance is optimal, the parameters for the three controllers are shown in Table 2.

**Figure 3:**
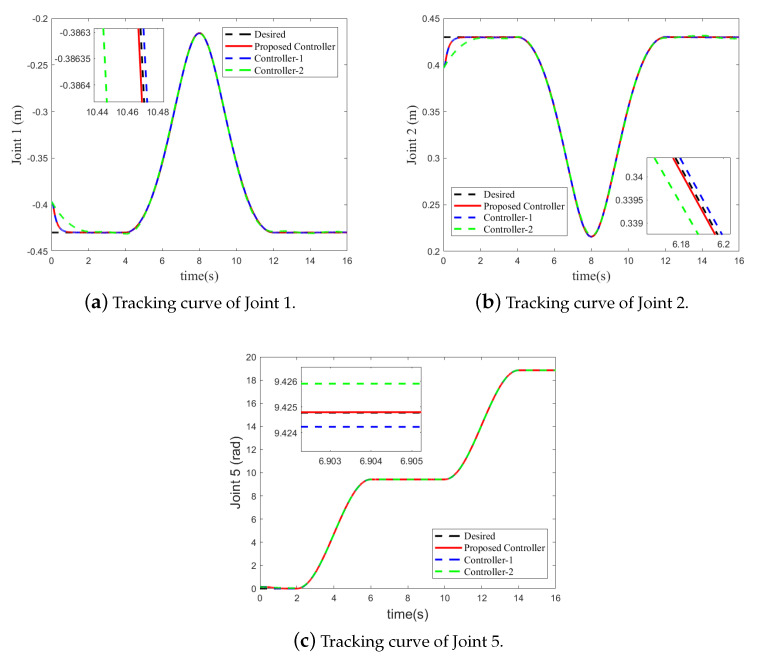
Tracking curves of the unilateral active joints under the three controllers.

**Figure 4:**
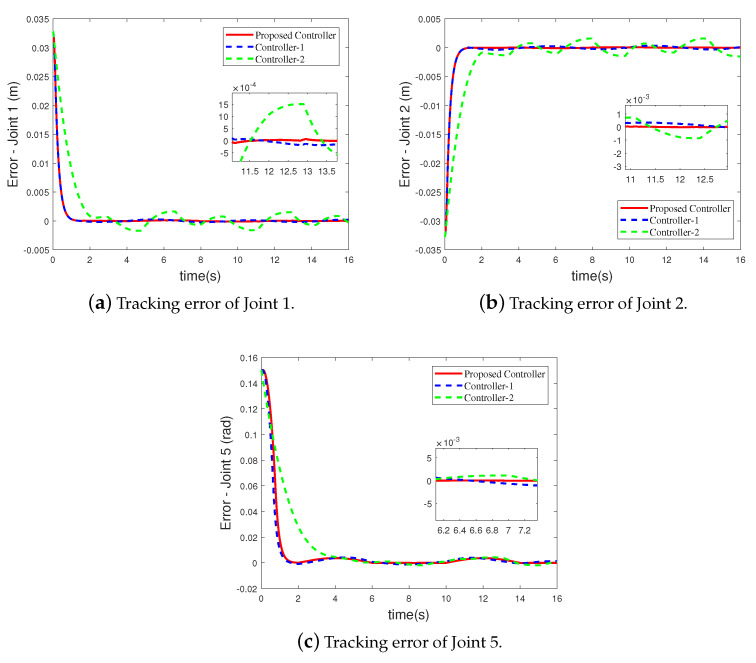
Tracking error curves of the unilateral active joints under the three controllers.

**Figure 5:**
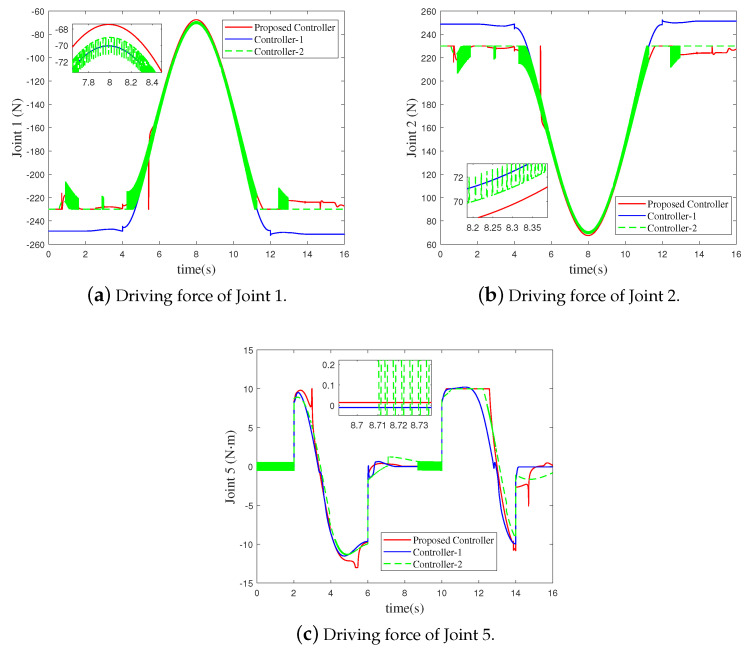
Driving force curves of the unilateral active joints under the three controllers.

**Figure 6:**
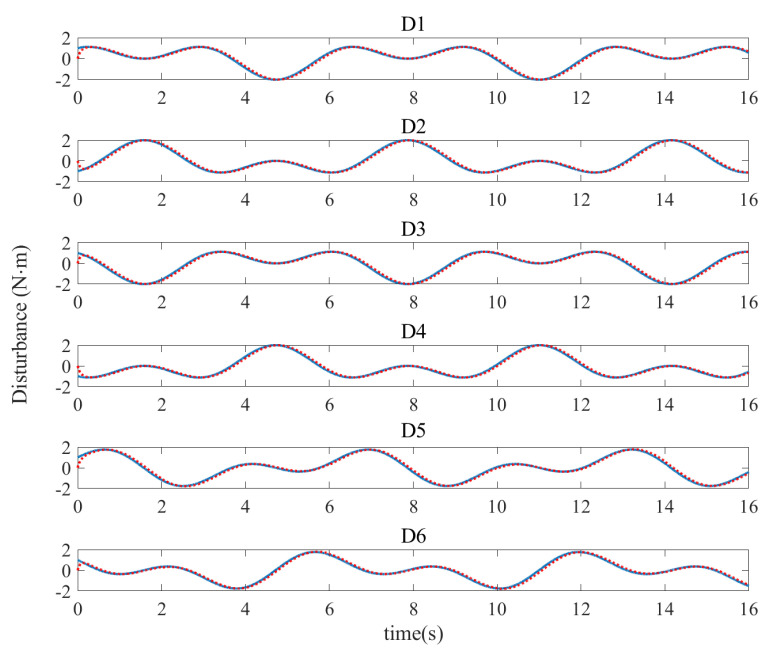
Estimation of the disturbance under the disturbance observer. (Blue: the actual disturbance; Red: the estimated disturbance.)

Since the bilateral symmetrical structure of the CPR, only the simulation results of the three active joints (Joint 1, Joint 2 and Joint 5) on one side of the CPR are depicted in Figure 3, Figure 4, Figure 5 and Figure 6. Under the three controllers, the tracking curves and tracking error curves of the unilateral active joints are illustrated in Figure 3 and Figure 4, respectively. The driving forces of the active joints are depicted in Figure 5. The result of the disturbance observation is shown in Figure 6. Table 3 presents the maximum steady-state error data of each active joint and the root mean square error data (RMSE) under the three controllers. The settling time of each active joints under the three controllers are listed in Table 4.

It can be revealed from Figure 3 and Figure 4 that compared to Controller-2, the tracking performance of the CPR is better under the proposed FT-NTSMC. This is because the finite-time dynamic tracking control can be achieved by the proposed controller based on the novel auxiliary system (12). As analyzed in Remark 1, however, when dealing with the input saturation problem, Controller-2 based on the existing auxiliary system (4) only achieves the asymptotic tracking performance. It should be noted that although the tracking performance under Controller-1 is similar to that of the proposed FT-NTSMC scheme, input saturation is not considered by Controller-1. Moreover, the maximum steady-state error data and RMSE data in Table 3 shows the excellent tracking performance of the proposed controller. The settling time data listed in Table 4 shows that the tracking errors of the active joints can converge to the origin at a fast speed under the proposed controller.

As illustrated in Figure 5, the actual control torques of the proposed FT-NTSMC are limited within [−230,0]N for Joint 1 and Joint 3, [0,230]N for Joint 2 and Joint 4, and [−13,10]N·m for Joint 5 and Joint 6. However, the control torques of Controller-1 obviously violate the constraints. Moreover, the chattering of FT-NTSMC is significantly reduced than that of the Controller-2, which has been explained in Remark 5. An observation of Figure 6 reveals that the designed disturbance observer in (8) provides good performance in estimating the disturbances.

Therefore, the validity and practicability of the proposed FT-NTSMC scheme are attested by the above analysis of the simulation results.

## 5. Experimental Results

**Figure 7:**
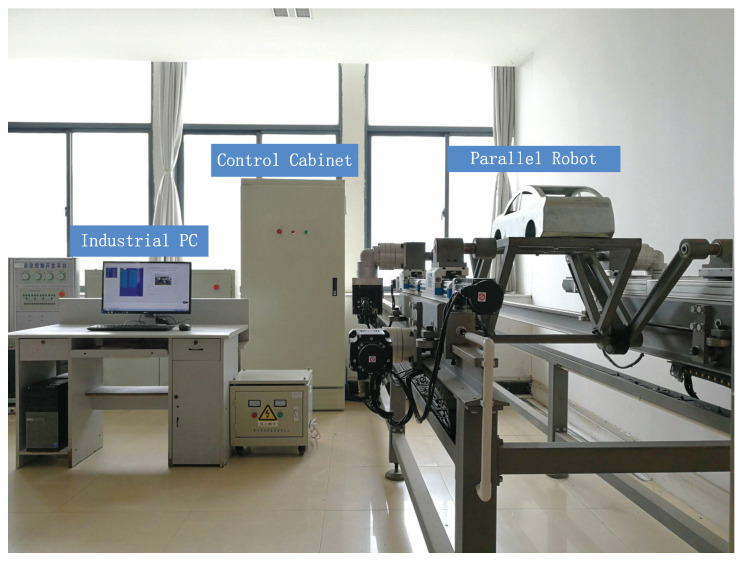
The prototyping system of the CPR.

In this section, relying on the experimental platform of the 3-DOF CPR depicted in Figure 7, the superiority of the developed FT-NTSMC for parallel robots is attested experimentally.

**Figure 8:**
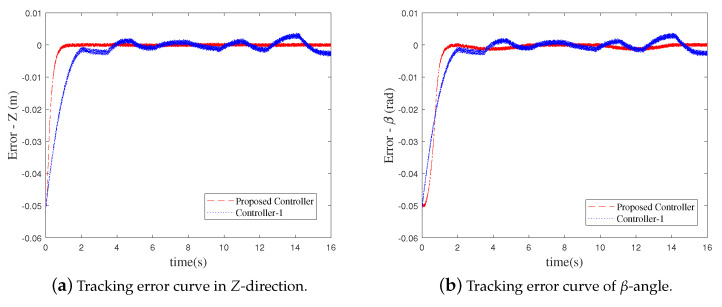
The experimental results of the tracking errors of the end-effector.

Since the control performance of the proposed FT-NTSMC and Controller-1 are better as shown in the simulation results, the two comparative control methods are experimentally evaluated based on the prototyping system of the CPR. As for the CPR, the posture components in *Z*-direction and β-angle exist during the motion process of the end-effector. The tracking error curves of the end-effector are shown in Figure 8. The maximum steady-state errors of the end-effector are listed in Table 5.

As exhibited in Figure 8 and Table 5, compared to Controller-1, the convergence speed and the tracking precision are higher when the developed FT-NTSMC is applied to the CPR. It should be noted that although the tracking performance of Controll-1 is similar to that of the proposed FT-NTSMC in the simulation results, input saturation is not imposed on Controller-1 in the simulation. Due to the physical limitations of actuators in the actual CPR system, input saturation is inevitable. Thus, the control performance of Controller-1 is degraded. By contrast, thanks to the disturbance observer (8) and the novel finite-time auxiliary system (12), the control performance of the proposed FT-NTSMC is still outstanding in the presence of uncertainties and input saturation.

The experimental results above further validate the high control performance of the proposed FT-NTSMC for parallel robots with uncertainties and input saturation.

## 6. Discussion

The finite-time dynamic tracking control for parallel robots with uncertainties and input saturation is investigated in the paper. The disturbance observer, the novel finite-time convergent auxiliary system and the NTSMC algorithm are incorporated to propose the FT-NTSMC scheme for parallel robots. To be specific, the disturbance observer is designed to estimate the lumped disturbance in the parallel robot system. By introducing the fractional exponential powers into the existing asymptotic convergent auxiliary system, the novel finite-time convergent auxiliary system is constructed to compensate for input saturation. The FT-NTSMC is proposed based on the disturbance estimation and the state of the novel auxiliary system. Two comparison control methods are also developed in the numerical simulation, i.e., Controller-1 and Controller-2. Compared to Controller-1, the input saturation is solved by the proposed FT-NTSMC. Compared to Controller-2, the finite tracking control of parallel robots is realized, which is helpful to enhance the control precision, convergence speed and robustness. These advantages of the FT-NTSMC over Controller-1 and Controller-2 are also analyzed in the simulation and experiment. Moreover, the computational burden of the proposed control algorithm is increased compared with Controller-1 and Controller-2, but it is still within a reasonable range.

In practical application, the proposed FT-NTSMC scheme can be applied to industry context, such as the CPR used for automobile electro-coating. The finite-time tracking control of the CPR is of great meaning to improve the quality of electrophoretic coating. Since it is random and time-consuming to adjust the parameters of the designed controller by experience, the parameter optimization problem should be addressed in the controller design for parallel robots. In future work, optimizing the parameters of the designed controller via the particle swarm optimization algorithm will be a potential direction, so that the control performance can be further improved by selecting the optimal parameters.

## 7. Conclusions

The FT-NTSMC is proposed for the finite-time dynamic tracking control of parallel robots with uncertainties and input saturation. A disturbance observer is designed to estimate the lumped disturbance in system. By introducing the fractional exponential powers, a novel auxiliary system with finite-time convergence is constructed to compensate for input saturation. Thus, the proposed controller consists of three parts, i.e., a NTSMC term based on the disturbance estimation to compensate for the lumped disturbance; an auxiliary variable-based control term to compensate for the input saturation and an equivalent control term. The finite-time stability of the resulting closed-loop system is proved with Lyapunov theory. The superiority of the proposed scheme is illustrated by comparative simulations and experiment on a 3-DOF CPR.

## Figures and Tables

**Table 1:** Physical parameters of the CPR.

Parameter	Value
Connecting rod length $l_{1}=l_{2}=l_{3}=l_{4}$	0.495 m
Diameter of gear dividing circle *d*	0.0344 m
Radius of driving wheel $r_{1}$	0.075 m
Radius of driving wheel $r_{2}$	0.025 m
Connecting rod length $l_{7}$	0.72 m
Reduction ratio of reducer	1:20
Screw lead *s*	0.01 m
Base length	1 m

**Table 2:** Parameters of the three controllers for the CPR.

Controllers	Parameters
FT-NTSMC	$\beta =I_{6\times 6},\alpha =1.5,L=100\times \mathrm{diag}\left(5,5,5,5,6,6\right)$,
	$\gamma_{1}=0.5,\gamma_{2}=1,\gamma_{3}=0.02,p_{1}=3,q_{1}=5$,
	$p_{2}=4,q_{2}=3,\zeta_{0}=0.05,k_{0}=10^{-5},k_{1}=10^{-4}$,
	$K_{2}=2I_{6\times 6},K_{3}=\mathrm{diag}\left(2,2,2,2,3,3\right)$.
Controller-1	$\beta =I_{6\times 6},\alpha =1.5,k_{0}=0.001$,
	L=100×diag(5,5,5,5,6,6).
Controller-2	$A=I_{6\times 6},\zeta_{0}=0.05,K=\mathrm{diag}\left(1,1,1,1,1.3,1.3\right)$,
	$B=0.5I_{6\times 6},P=\mathrm{diag}\left(0.1,0.1,0.1,0.1,0.3,0.3\right)$.

**Table 3:** Maximum steady-state error of the unilateral active joints and the RMSE under the three controllers.

	FT-NTSMC	Controller-1	Controller-2
Joint 1	$1.1521\times 10^{-4}\;m$	$2.7395\times 10^{-4}\;m$	$1.6\times 10^{-3}\;m$
Joint 2	$8.3279\times 10^{-5}\;m$	$3.436\times 10^{-4}\;m$	$1.6\times 10^{-3}\;m$
Joint 5	$3.7\times 10^{-3}\;\mathrm{rad}$	$4.2\times 10^{-3}\;\mathrm{rad}$	$4.6\times 10^{-3}\;\mathrm{rad}$
RMSE	$2.37\times 10^{-4}$	$2.41\times 10^{-4}$	$3.19\times 10^{-3}$

**Table 4:** The settling time of the unilateral active joints under the three controllers.

	FT-NTSMC	Controller-1	Controller-2
Joint 1	0.9s	0.9s	2.1s
Joint 2	0.9s	0.9s	2.1s
Joint 5	1.3s	1.3s	3.8s

**Table 5:** Maximum steady-state errors of the end-effector.

	FT-NTSMC	Controller-1
*Z*	$7.025\times 10^{-4}\;m$	$3.762\times 10^{-3}\;m$
β	$6.32\times 10^{-4}\;\mathrm{rad}$	$3.777\times 10^{-3}\;\mathrm{rad}$

## Data Availability

Not applicable.
